# Acupuncture ameliorates Mobile Phone Addiction with sleep disorders and restores salivary metabolites rhythm

**DOI:** 10.3389/fpsyt.2023.1106100

**Published:** 2023-02-21

**Authors:** Hong Yang, Kun Yang, Lu Zhang, Ni Yang, Ying-Xiu Mei, Ya-Li Zheng, Yan He, Yan-Ju Gong, Wei-Jun Ding

**Affiliations:** ^1^School of Medical and Life Sciences, Chengdu University of Traditional Chinese Medicine, Chengdu, China; ^2^Department of Fundamental Medicine, Chengdu University of Traditional Chinese Medicine, Chengdu, China

**Keywords:** salivary metabolites, circadian, dysrhythmia, Mobile Phone Addiction, sleep disorder, acupuncture

## Abstract

**Objectives:**

Mobile Phone Addiction (MPA) is a novel behavioral addiction resulting in circadian rhythm disorders that severely affect mental and physical health. The purpose of this study is to detect rhythmic salivary metabolites in MPA with sleep disorder (MPASD) subjects and investigate the effects of acupuncture.

**Methods:**

Six MPASD patients and six healthy controls among the volunteers were enrolled by MPA Tendency Scale (MPATS) and Pittsburgh Sleep Quality Index (PSQI), then the salivary samples of MPASD and healthy controls were collected every 4-h for three consecutive days. Acupuncture was administered for 7 days to MPASD subjects, then saliva samples were collected again. Salivary metabolomes were analyzed with the method of LC-MS.

**Result:**

According to our investigation, 70 (57.85%) MPA patients and 56 (46.28%) MPASD patients were identified among 121 volunteers. The symptoms of the 6 MPASD subjects were significantly alleviated after acupuncture intervention. The number of rhythmic saliva metabolites dropped sharply in MPASD subjects and restored after acupuncture. Representative rhythmic saliva metabolites including melatonin, 2′-deoxyuridine, thymidine, thymidine 3′,5′-cyclic monophosphate lost rhythm and restored after acupuncture, which may attribute to promising MPASD treatment and diagnosis biomarkers. The rhythmic saliva metabolites of healthy controls were mainly enriched in neuroactive ligand-receptor interaction, whereas polyketide sugar unit biosynthesis was mainly enriched in MPASD patients.

**Conclusion:**

This study revealed circadian rhythm characteristics of salivary metabolites in MPASD and that acupuncture could ameliorate MPASD by restoring part of the dysrhythmia salivary metabolites.

## Introduction

Mobile Phone Addiction (MPA) is a behavioral epidemic impacting millions of adults and adolescents worldwide. MPA, also named mobile phone abuse, problematic mobile phone use, or “nomophobia,” is caused by overuse or addiction to the smartphone. Like behavioral addictions such as gambling and shopping addiction, MPA is closely associated with insecurity, anxiety and depression, staying late, poor parent-child relationships, leisure boredom, and psychological disorders (1). College students who played games for more than 2 h per day usually had later bedtime, poorer sleep quality, and higher daytime sleepiness (2). Because mobile phones are an essential part of adolescents’ lives in the era of the popularized Internet, the incidence of MPA among college students is blooming globally. For instance, 16.9% of Swiss students were identified as MPA (3). A Chinese cross-sectional study showed that 29.8% of college students were MPA subjects (4). The incidence of MPA has dramatically increased during the COVID-19 pandemic (5). Hence, MPA among adolescents needs urgent attention (6, 7).

Mobile Phone Addiction is characterized by circadian dysrhythmia syndrome, including sleep disorders. The circadian clock is ubiquitously presented to almost all life forms on earth. A master clock in the brain dominates all peripheral clocks to keep the body in sync. Circadian rhythm works through sophisticated physiological reactions, including genes, proteins, lipids and metabolites, resulting in the diurnal rhythmicity of circadian metabolites (8). Circadian clock genes have also been connected to neuro-behavioral problems like addiction to alcohol and drugs, sleep difficulties, and other psychiatric illnesses. In the investigation of the effects of individual circadian rhythm differences on insomnia, impulsivity, and food addiction, later in the day (evening type) circadian preferences were indirectly associated with higher food addiction scores mediated by insomnia and impulsivity (9). Accumulating literature indicates that sleep disorders are one of the unpleasant side effects of MPA (10–12). MPA with sleep disorders (MPASD) usually induces migraine (or tension headaches) and retinal damage (13–15). Sleep is the key regulator of circadian rhythmicity, and misaligned sleep causes circadian disorders (16, 17). Currently the majority of MPASD studies focused on frequency, duration, and usage at bedtime or after light out (18, 19). Overexposed blue light late at night disrupts the rhythmicity of nocturnal hormonal secretions such as melatonin and cortisol (20), resulting in circadian dysrhythmia in MPASD subjects. Because the concentration of human metabolites is also regulated by the circadian clock, which exhibits oscillation, studying the characteristics of metabolite rhythms and sampling at the appropriate time is more conducive to accurate biomarker screening.

Acupuncture is considered an effective intervention to restore circadian dysrhythmia in MPASD patients. Acupuncture, as a part of traditional Chinese medicine, has shown its effectiveness for a variety of disorders, including insomnia, complex psychological problems (21) and behavioral addiction (22, 23). Zhang reported that electro-acupuncture and psychological intervention could significantly improve impulsive behavior among internet addiction adolescents through increasing N-acetyl aspartate (NAA) and choline (Cho) levels in prefrontal and anterior cingulate cortices (24). Wang’s findings also verified the modulation effect of acupuncture on functional connectivity of reward and habit systems related to the ventral striatum in internet addition individuals (25). Clinical observations showed that acupuncture could be helpful for circadian disorders by regulating the rhythmicity of blood pressure in hypertension patients (26), restoring the function of the autonomic nervous system in night-shift workers (27), and rebalancing the sympathetic and parasympathetic activities in night-shift individuals (28).

MPASD subjects have rarely been evaluated for dysrhythmic salivary metabolites, let alone for acupuncture interventions and promising biomarkers. In this work, MPASD as a representative MPA subtype was selected. Saliva, one of the most accessible body fluids, was used to reveal diurnal metabolic oscillations (29, 30). The MPATS and PSQI were used for quantitative analysis of acupuncture effects. By means of LC/MS, dysrhythmic salivary metabolites and potential markers were determined to reveal pathological mechanisms of MPASD.

## Materials and methods

### Recruitment of MPASD subjects

This work was performed in compliance with the Declaration of Helsinki and was approved by the Ethics Committee of the Affiliated Hospital, Chengdu University of Traditional Chinese Medicine (NO: 2021KL-094). A WeChat mini-program was used to execute questionnaires for selecting volunteers in Chengdu, China. All participants provided written informed consent before the study. MPASD volunteers and healthy controls were recruited by the MPATS and PSQI. The MPATS contains 16 questions, including withdrawal symptoms, salience, social comfort, and mood changes. The inclusion criteria of MPASD subjects were ≥ 40 (MPATS) and ≥ 7 (PSQI) scores (31). To minimize the interference of severe sleep disorders upon MPA, those characterized as “poor” sleepers (7 ≥ PSQI ≥ 15 scores) were recruited from a non-clinical population (32). The PQSI score was made up of questions including sleep quality, sleep latency, sleep duration, sleep efficiency, sleep disturbance, daytime dysfunction, and medication use. The exclusion criteria included one of the following conditions: (1) Oral diseases, including periodontitis, bleeding gums or tooth decay. (2) The antibiotic regimen within the last 3 months. (3) Upper respiratory tract infection or rhinitis or pharyngitis whinth 1 month. (4) Smokers and alcohol abusers and other types of addicts. Healthy controls were simultaneously recruited into group N. All enrolled subjects completed the 72-h constant routine.

### Acupuncture intervention

Six MPASD subjects were selected randomly into group M and received acupuncture on Baihui (GV20), Shenting (GV24), Yintang (GV29), bilateral Anmian (EX-HN22), bilateral Hegu (LI 4), bilateral Neiguan (PC6), bilateral Shenmen (HT7), and bilateral Sanyinjiao (SP6) (21, 24). Acupuncture needles were inserted to a depth of 17–25 mm. Rotating or lifting manipulation was used to obtain the “deqi” sensation for 30 min once a day and lasted for 7 d. Those who received acupuncture treatment were included in group T.

### Sample collection and preparation

**FIGURE 1:**
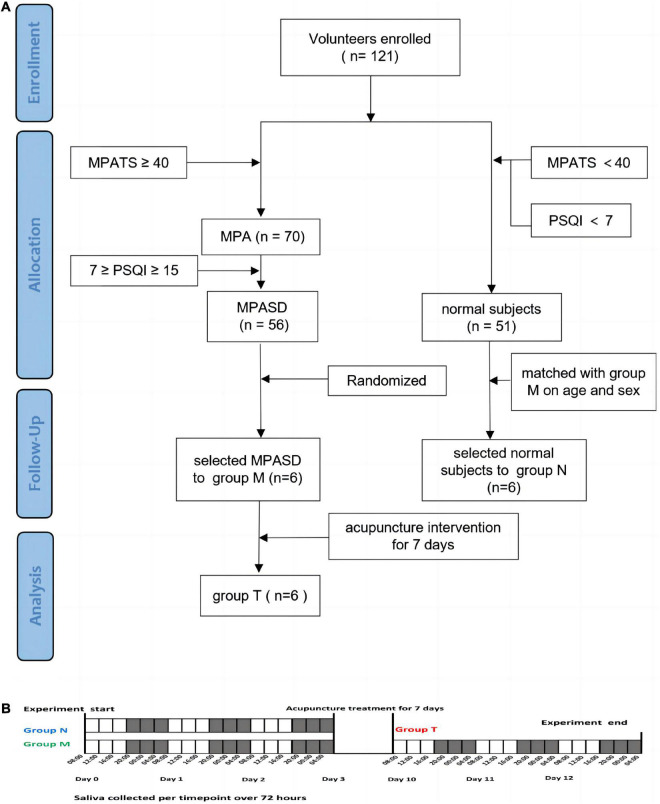
Schematic diagram of group enrollment and sample collection. Flow chart of the experimental design **(A)**. Saliva collection protocol **(B)**.

All subjects in the three groups salivary samples were collected in the following procedure (Figure 1). The unstimulated saliva samples (∼3 ml) were collected by spitting into clear tubes at 4-h intervals (08:00, 12:00, 16:00, 20:00, 00:00, and 04:00) for a period of three consecutive days (number of samples per subject, *n* = 18; the total number of salivary samples, *n* = 324). All volunteers were prohibited from cleaning their mouths or eating 2 h before sampling. To minimize diet interference, all participants took the same meals prepared based on the Guidelines for Chinese Residents (2021). All participants were awakened no more than 5 min before 0:00 or 4:00 for sampling, while there was no additional light exposure. The collected samples were immediately placed on ice and centrifuged for 15 min at 3000 rpm. To reduce the sampling errors within individuals, every two subjects, respectively, in group N and group M were randomly (33) selected as a mixed one subject, then their samples of the same time for 3 days were mixed as one sample (i.e., the mixture sample of 08:00 was equally mixed by the three successive days sampled at 08:00 of two subjects from the same group). The samples from group T were mixed in the same way as group M. The mixed supernatants were transferred into a clean tube and stored at −80°C.

### LC-MS analysis and data processing

The frozen supernatants of saliva samples (∼1.5 mL) were thawed at room temperature, centrifuged at 15,000 g and 4°C for 3 min, and resuspended in prechilled 80% methanol and 0.1% formic acid. The samples were then incubated on ice for 5 min and centrifuged at 15,000 g and 4°C for 20 min. The supernatants were diluted to 53% methanol with LC-MS grade water, transferred to a fresh tube and centrifuged at 15,000 g and 4°C for 20 min. Finally, the supernatants were injected into a UHPLC–MS/MS system (ThermoFisher) for metabolome analysis.

The data were assessed by a Nexera UPLC system (Shimadzu Corporation, Japan) coupled with a Q-Exactive quadrupole-Orbitrap mass spectrometer (ThermoFisher, MA, USA). The raw data were evaluated by Progenesis QI V2.3 software (Non-linear, Dynamics, Newcastle, UK). The Human Metabolome Database (HMDB), Lipidmaps (V2.3), Metlin, EMDB, and PMDB were used to perform qualitative compound identification. The extracted data were further processed by removing any peaks with a missing value in more than 50% of groups, replacing the zero value with half of the minimum value, and screening the qualitative results of the compound. Compounds with resulting scores below 36 points were considered to be inaccurate and thus removed. The screening criteria of differential metabolites were variable important in projection (VIP) value > 1 and *p* < 0.05 for the *t* test and the Benjamin Hochberg calculation to control the multiple false results. The metabolic functions and pathways were enriched by MBROLE 2.0.^1^

### Determination of the circadian rhythmicity of salivary metabolites

The JTK_CYCLE package was used to assess the circadian rhythm in salivary metabolites,^2^ which provided phase (LAG), amplitude (AMP), and period (PER) estimates and adjusted *p* values based on metabolite expression at six-time points of sampling. An adjusted *p* < 0.05 was identified as a metabolite with diurnal rhythmicity (34). To visualize the rhythmically expressed metabolites, the metabolite expression levels were linearly normalized by the formula $x^{\prime }=\log10\left(x\right)$ where *x*′ represents the normalization results of metabolite expression and *x* represents the metabolite expression at each time point.

### Software and statistical analysis

The data analysis and visualizations were performed by GraphPad Prism 9 (La Jolla, USA) and visualization software Venn.^3^ Student’s *t* test and fold change analysis were used to compare the differentially expressed metabolites between groups. ANOVA analysis was used to compare the three groups’ data. Heatmaps were generated using online software.^4^

## Results

### Characteristics of the enrolled volunteers

A total of 121 volunteers, 65 females and 56 males and aged 18–29 years, were enrolled in the questionnaire investigation. Of these volunteers, 70 (57.85%) met the MPA diagnostic criteria, and 56 (46.28%) were MPASD patients. Six MPASD patients, aged 21∼29 years and with BMI of 19.6∼22.3, were randomly included in group M. Six age and sex-matched healthy volunteers (group N) were simultaneously recruited. The average scores of both the MPATS and PSQI of group M were significantly higher than those of group N (Table 1). According to Driller’s definition (32), those included in group N were “good” sleepers, whereas those in group M were “poor” sleepers.

**TABLE 1:** Essential characteristics of the recruited subjects.

Characteristics	Group N	Group M	*p*
Age	24.50 ± 3.271	24.67 ± 3.011	0.9287
BMI	21.02 ± 0.966	20.82 ± 1.155	0.7516
MPATS scores	31.50 ± 2.258	48.00 ± 7.772	0.0005***
PSQI scores	3.167 ± 2.137	10.33 ± 2.422	0.0005***

***p < 0.001.

### Acupuncture intervention ameliorate MPASD

The average MPATS scores of subjects in group T were markedly lower after acupuncture intervention for 7 d, and withdrawal symptoms were also significantly restored (Table 2). The average PSQI scores in group M were much higher than those in group T, showing that acupuncture effectively alleviated the main symptoms of MPASD patients, particularly sleep quality and sleep latency (Table 2).

**TABLE 2:** MPATS and PSQI scores before and after acupuncture intervention.

Item	Group M	Group T	*p*
MPATS scores	48.00 ± 7.772	39.83 ± 4.167	0.0467*
Withdrawal symptoms	21.50 ± 3.082	17.17 ± 1.169	0.0092**
PSQI scores	10.33 ± 2.422	4.833 ± 1.169	0.0005***
Sleep quality	2.833 ± 0.408	0.6667 ± 0.516	<0.0001***
Sleep latency	2.000 ± 0.633	1.000 ± 0.633	0.0209*
Daytime dysfunction	1.500 ± 0.837	0.333 ± 0.516	0.0157*

*p < 0.05; **p < 0.01; ***p < 0.001.

### Saliva metabolites change in overall levels

**FIGURE 2:**
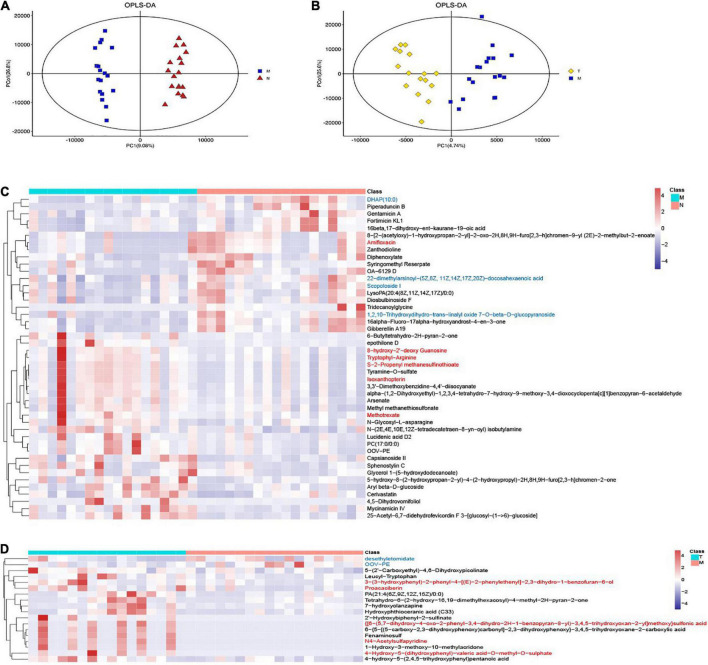
OPLS-DA and heatmaps show differentially expressed saliva metabolites. The OPLS-DA images indicated differentially expressed metabolites in MPASD patients **(A)** and after acupuncture intervention **(B)** heatmaps show the differentially expressed saliva metabolites in MPASD patients **(C)**, and after acupuncture intervention **(D)** the top VIP significantly upregulated (red) and downregulated (blue) metabolites.

The saliva metabolites between groups N and M were significantly different according to OPLS-DA. Compared with healthy controls, 45 differentially expressed metabolites were detected in the saliva samples (Figures 2A, C). The top differential metabolites were shown in the heatmap, the significantly regulating metabolites included tyramine-O-sulfate, S-2-propenyl methanesulfinothioate, scopoloside i, DHAP (10:0), etc. Pathways involved in the differential metabolites were enriched in choline metabolism in cancer and glycerophospholipid metabolism (Supplementary Table 1).

On the other hand, acupuncture intervention rescued some abnormally expressed salivary metabolites in MPASD patients. Eighteen differentially expressed metabolites were identified by OPLS-DA (Figure 2B), The top differential metabolites were shown in the heatmap and significantly regulating metabolites, including 4-hydroxy-5-(dihydroxyphenyl)-valeric acid-O-methyl-O-sulphate, 3-(3-hydroxyphenyl)-2-phenyl-4-[(E)-2-phenylethenyl]-2,3-dihydro-1-benzofuran-6-ol, proacaciberin, desethyletomidate, 1-hydroxy-3-methoxy-10-methylacridone, OOV-PE. Besides, the enriched differential metabolite pathways after acupuncture were enriched in Tryptophan metabolism (Supplementary Table 1).

### Salivary metabolites with circadian dysrhythmia in MPASD

**FIGURE 3:**
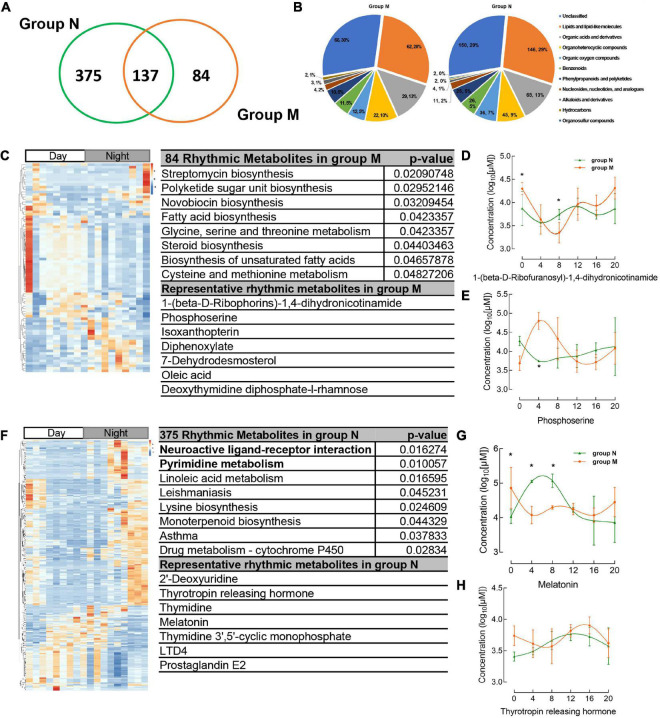
Circadian rhythmic metabolites were identified from salivary samples. **(A)** The number of saliva metabolites with circadian rhythm (adj. *p* < 0.05) is shown as a Venn diagram. **(B)** The major classes of salivary metabolites with circadian rhythm. **(C)** Heatmap of 84 rhythmic metabolites which show only rhythm in group M and their KEGG enrichment pathway, representative metabolites in the chart. **(D,E)** The concentration of representative metabolites 1-(beta-D-ribophorins)-1,4-dihydronicotinamide, phosphoserine. **(F)** The metabolites which disappear rhythmicity in MPASD mean that only rhythmic in group N and KEGG enrichment and representative metabolites. **(G,H)** The concentration of representative rhythmic metabolites melatonin and thyrotropin releasing hormone. *p < 0.05.

There were 512, 221 salivary metabolites with circadian rhythmicity detected in groups N and M, respectively, (Supplementary Table 2), and visually showed 596 rhythmic metabolites in a Venn diagram (Figure 3A and Supplementary Table 3). These rhythmic metabolites were primarily classified into lipids and lipid-like molecules, organic acids, and derivatives (Figure 3B and Supplementary Table 4). 84 rhythmic metabolites showed rhythm only in group M and were enriched in pathways including streptomycin biosynthesis, polyketide sugar unit biosynthesis, Novobiocin biosynthesis, Fatty acid biosynthesis, etc., (Figure 3C and Supplementary Table 5), the representative metabolites including 1-(beta-D-Ribophorins)-1,4-dihydronicotinamide, phosphoserine (Figure 3C). The representative metabolites concentration in group M and group N were shown (Figures 3D, E). While, 375 metabolites were shown circadian rhythmic disappearance in MPASD subjects, and only rhythmic in healthy subjects. By KEGG enrichment, the 375 metabolites were in neuroactive ligand receptor interaction, pyrimidine metabolism, linoleic acid metabolism. And the representative metabolites include thyrotropin releasing hormone, melatonin (Figures 3F–H and Supplementary Table 5).

**FIGURE 4:**
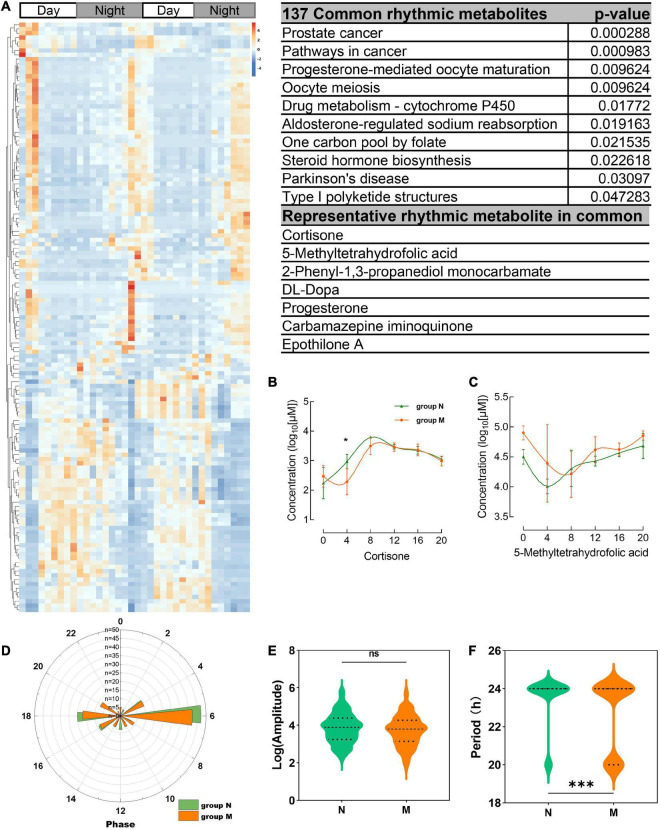
Common shared rhythmic metabolites in group N and group M were identified from salivary samples. **(A)** The heatmap of common saliva metabolites with circadian rhythm and KEGG enrichment pathway and representative metabolites were shown. **(B,C)** The concentration of representative common rhythmic metabolites cortisone, 5-methyltetrahydrofolic acid. **(D–F)** The disruption phrase, amplitude and period of common shared rhythmic metabolites. *p < 0.05.

137 salivary metabolites were shown common rhythmic both in group M and group N and KEGG enrichment and representative metabolites (Figures 4A–C). The parameters of the common metabolites were compared between the two groups, indicating that only the period of the metabolites showed significant differences, while the amputation and disruption of phrase didn’t display significant changes (Figures 4D–F and Supplementary Table 5).

### Acupuncture intervention restored salivary metabolites in dysrhythmic

**FIGURE 5:**
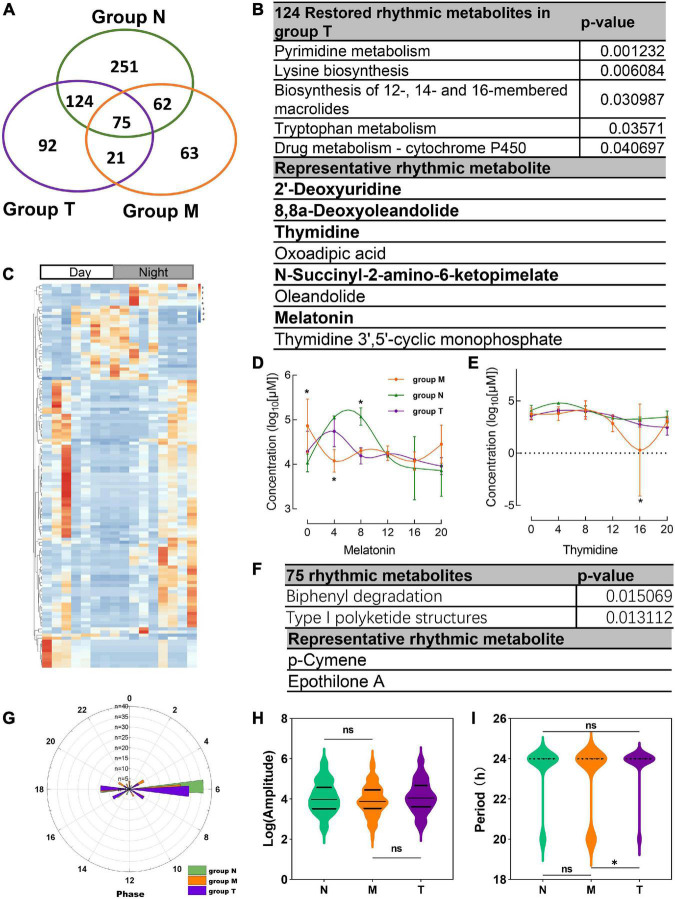
Acupuncture restored rhythmic saliva metabolites and shared rhythmic metabolites analysis. **(A)** Venn diagram showing 688 rhythmic metabolites in three groups. **(B)** The KEGG pathway enrichment of acupuncture restored rhythmic salivary metabolites and representative metabolites. **(C)** Heatmap of 124 rhythmic metabolites in group T. The concentration of representative metabolites melatonin **(D)**, thymidine **(E)**. **(F)** Common shared metabolites in three groups enrichment and representative metabolites. **(G)** The circadian parameter of common shared metabolites in three groups phrase distribution. **(H,I)** The amplitude and period of the three groups. *p < 0.05.

312 salivary metabolites with circadian rhythmicity were detected in group T and visually showed 688 rhythmic metabolites in a Venn diagram (Figure 5A). According to the Venn diagram, 124 metabolites were restored rhythmic in group T, namely, the MPASD subjects who received acupuncture, a heatmap was used to show the metabolites (Figures 5B, C and Supplementary Table 3). Further, the salivary metabolites with special rhythmicity were analyzed by the KEGG database. The results showed significant pathways, including enriched in pyrimidine metabolism, lysine biosynthesis. Interesting, the circadian rhythm of several vital metabolites, including melatonin, thymidine, 2′-deoxyuridine, 8,8a-deoxyoleandolide oxoadipic acid, N-succinyl-2-amino-6-ketopimelate, oleandolide, thymidine 3′,5′-cyclic monophosphate disappeared in MPASD patients; however, they were restored after acupuncture intervention (Figures 5B, D, E and Supplementary Table 5).

Finally, the 75 rhythmic saliva metabolites shared by the three groups were analyzed by the KEGG database. The results showed the pathways, including biphenyl degradation, type I polyketide structures pathway could be the common pathway, and p-cymene, epothilone A were the representative metabolites (Figure 5F). The parameter of the circadian rhythmic analysis found that there were no significant differences in amplitude and phase distribution among the three groups (Figures 5G, H). But the period dropped significantly in group T compared with group N and M (Figure 5I).

## Discussion

Our work observed a relatively high incidence of MPASD. Our investigation found that enrolled volunteers were estimated to be 57.85% of MPA and 46.28% of MPASD, both higher than the prevalence of Chinese undergraduates (36.6 and 33.2%, respectively) reported by a recent article (35). The present work aimed to screen potential MPASD volunteers, not a classical cross-sectional questionnaire-based survey or stratified random sampling method, resulting in markedly higher ratios of MPA and MPASD subjects than those of conventional epidemiological investigations. Nevertheless, the alarmingly high prevalence of MPA among undergraduates is still a great challenge. MPA is a type of behavioral addiction similar to Internet addiction. Considering that circadian rhythm was intensively disrupted in insomnia or severe sleep disorder patients, poor sleepers (7 ≤ PQSI scores < 15) were selected to minimize the impacts of sleep disorders on MPA subjects. On the other hand, both MPATS and PSQI scores decreased significantly after acupuncture intervention. In particular, the withdrawal symptoms of MPASD subjects were greatly ameliorated. Previous studies reported that acupuncture could treat Internet addiction, possibly based on the patients’ reward and habit systems (25). The improved indexes mainly included sleep quality, sleep latency, and daytime dysfunction, indicating that acupuncture intervention significantly reduced the MPASD symptoms mentioned above.

Our work revealed in an overall level that there were significant metabolites altered like tryptophyl-arginine associated with the connectivity of critical cephalic regions for the extended reward network (36) showed significant upregulation in MPASD subjects and was enriched in the glycerophospholipid metabolism pathway in our results. Then, the enriched KEGG pathways (i.e., choline metabolism in cancer and glycerophospholipid metabolism) in MPASD patients may represent an underlying metabolic pathway involved in MPASD progression. Glycerophospholipid metabolism has been reported in a chronic alcohol-exposed rat model to regulate the alcohol abuse mechanism (37), whereas choline metabolism is regulated in Internet addiction (38).

Importantly, the diurnal dysrhythmic features of saliva metabolites in MPASD subjects. First, a considerable proportion of saliva metabolites derived from MPASD patients lost their daily rhythmicity. A total of 512 and 221 salivary metabolites with diurnal rhythmicity were identified in the healthy controls and MPASD patients, respectively; the disappearance of 291 (56.84%) kinds of rhythmic salivary metabolites indicated that MPASD extensively disturbed the circadian rhythmicity of salivary metabolites. These results were similar to Gehrman (39), who showed that in insomnia serum circadian metabolites, the rhythmic metabolites and percentage were significantly changed compared with healthy controls. Besides, our results found lipids and lipid-like molecules were the most notable circadian metabolites that were markedly different from previously reported amino acids (40). Salivary circadian metabolites, especially 1-(beta-D-ribofuranosyl)-1,4-dihydronicotinamide, phosphoserine, isoxanthopterin, diphenoxylate, 7-dehydrodesmosterol, oleic acid, deoxythymidine diphosphate-l-rhamnose were displayed only rhythmic in MPASD, while Diphenoxylate was reported in the withdrawal treatment of opium addicts (41). However, we haven’t found addiction or sleep-related disease in the others. Then, the enriched KEGG pathways (i.e., streptomycin biosynthesis, polyketide sugar unit biosynthesis, novobiocin biosynthesis, fatty acid biosynthesis, glycine, serine and threonine metabolism, steroid biosynthesis, biosynthesis of unsaturated fatty acids, cysteine, and methionine metabolism) in MPASD patients may represent an underlying metabolic pathway involved in MPASD circadian dysrhythmia progression. Polyketide sugar unit biosynthesis was predicted involved in the microbial function in alcohol-dependent rats (42). Some metabolites showed rhythm in the health subjects including melatonin and LTD4. While LTD4 was reported in attenuates the development of opioid dependence in a mouse model of naloxone-induced opioid withdrawal syndrome (43), it lost rhythm in MPASD subjects, which meant that some metabolites could be a biomarker for diseases. For example, melatonin as a biomarker of circadian dysregulation, concentrations differ significantly from those of MPASD patients at certain times, such as 0:00 and 4:00, because the peak serum concentrations at these two time points in the physiological state and saliva also show higher levels. For this trend, precisely sampling at night to detect the corresponding metabolite levels can be regarded as a sensitive diagnostic biomarker for the disease. Cortisone concentrations follow the diurnal rhythm of hypothalamus-pituitary-adrenal axis, which is the common rhythmic metabolite despite the MPASD. And our results found that the period (but not amplitude and phase) of shared rhythmic metabolites in groups was the most variable parameter in circadian rhythm. Our work probed the potential therapeutic markers of acupuncture intervention. For the first time, we showed that acupuncture could effectively intervene in MPASD subjects. Both the MPATS and PSQI scores decreased significantly after acupuncture for 7 d, particularly in sleep quality and other SD-dependent indexes. Notwithstanding further exploration and optimization is needed to prove acupoints and/or treatment regimens, our work primarily showed the potential effects of acupuncture for MPASD. OPLS-DA and heatmap analysis showed that MPASD subjects were identical to those in acupuncture-treated volunteers (Figure 2), suggesting shared targets of pathogenesis and acupuncture therapy for MPASD patients. Venn diagram showed that acupuncture could extensively restore dysrhythmic salivary metabolites, of which the circadian rhythmic saliva metabolites, i.e., melatonin, 2′-Deoxyuridine, 8,8a-deoxyoleandolide, thymidine, oxoadipic acid, N-succinyl-2-amino-6-ketopimelate, oleandolide, thymidine 3′,5′-cyclic monophosphate all present rhythmic in healthy controls, disappeared in MPASD patients, and reappeared after acupuncture intervention. This interesting synchronous pattern indicated a pool of therapeutic markers for MPASD. For instance, melatonin, as a classic circadian hormone, monitors the sleep/wake, and circadian rhythm (44, 45); our work indicates that melatonin, as a drug addiction-associated hormone, may participate in behavioral addictions, including MPASD (46) and may be a potential marker for monitoring the effect of acupuncture intervention. The KEGG enriched pathways of the restored rhythmic metabolites in group T indicate that Pyrimidine metabolism and Lysine biosynthesis would be the potential acupuncture invention process.

Some limitations exist in the work. It should be firstly noted that this study has examined only a tiny MPASD population. Given the marked personal diversities and multiple factors that might impact the sample collection, a larger sample size might be required for further MPDSD studies, even though collecting 18 saliva samples from each subject for circadian rhythmic research is a difficult and tedious task. The second limitation of this study was that the acupuncture regimen might not be the optimal intervention for MPASD therapy. The acupoints used in this work were based on our differentiation of Chinese medical syndromes, whereas the 7-day course of treatment was derived from our clinical experience; whether the acupoint selection or the method of acupuncture intervention regime, it still needs further exploration. Thirdly, we only observed dysrhythmic saliva metabolites and misaligned metabolic pathways associated with circadian rhythmicity, and the underlying mechanism will urgently be detected. Finally, salivary metabolites are derived from the tripartite interactions between salivary microbiota, salivary metabolome and host circadian clock; how host-microbe-metabolite orchestration takes place in MPDSD pathogenesis and acupuncture intervention is worthy of further in-depth research.

## Conclusion

Our work revealed the characteristics of salivary metabolites with circadian dysrhythmia in MPASD patients and probed the therapeutic markers of the acupuncture intervention with a focus on salivary metabolites, providing a novel approach for the exploration of the molecular underpinnings in MPASD subjects.

## Data availability statement

Data will be made available on request.

## Ethics statement

The studies involving human participants were reviewed and approved by the Ethics Committee of the Affiliated Hospital, Chengdu University of Traditional Chinese Medicine. The patients/participants provided their written informed consent to participate in this study.

## Author contributions

HY: visualization, data curation, and writing—original draft. KY: validation, data curation, and formal analysis. Y-LZ, LZ, Y-XM, NY, and HY: investigation and resources. Y-JG: methodology, project administration. W-JD: conceptualization, writing—review and editing, and funding acquisition. All authors contributed to the article and approved the submitted version.

## Footnotes

1
http://csbg.cnb.csic.es/mbrole2/

2
https://openwetware.org/wiki/HughesLab:JTK_Cycle

3
http://www.ehbio.com/test/venn

4
https://www.oebiotech.com/

## References

[1] Sahu M Gandhi S Sharma M. Mobile phone addiction among children and adolescents: a systematic review. *J Addict Nurs.* (2019) 30:261–8. 31800517 10.1097/JAN.0000000000000309

[2] Akçay D Akçay B. The effect of computer game playing habits of university students on their sleep states. *Perspect Psychiatr Care.* (2020) 56:820–6. 10.1111/ppc.12497 32163182

[3] Haug S Castro R Kwon M Filler A Kowatsch T Schaub M. Smartphone use and smartphone addiction among young people in Switzerland. *J Behav Addict.* (2015) 4:299–307. 10.1556/2006.4.2015.037 26690625 PMC4712764

[4] Chen B Liu F Ding S Ying X Wang L Wen Y. Gender differences in factors associated with smartphone addiction: a cross-sectional study among medical college students. *BMC Psychiatry.* (2017) 17:341. 10.1186/s12888-017-1503-z 29017482 PMC5634822

[5] Marengo D Angelo Fabris M Longobardi C Settanni M. Smartphone and social media use contributed to individual tendencies towards social media addiction in Italian adolescents during the COVID-19 pandemic. *Addict Behav.* (2022) 126:107204. 10.1016/j.addbeh.2021.107204 34875508

[6] Stevens M Dorstyn D Delfabbro P King D. Global prevalence of gaming disorder: a systematic review and meta-analysis. *Aust N Z J Psychiatry.* (2021) 55:553–68. 10.1177/0004867420962851 33028074

[7] Mei S Chai J Wang S Ng C Ungvari G Xiang Y. Mobile phone dependence, social support and impulsivity in Chinese university students. *Int J Environ Res Public Health.* (2018) 15:504. 10.3390/ijerph15030504 29533986 PMC5877049

[8] Sato T Sassone-Corsi P. Nutrition, metabolism, and epigenetics: pathways of circadian reprogramming. *EMBO Rep.* (2022) 23:e52412. 10.15252/embr.202152412 35412705 PMC9066069

[9] Kandeger A Selvi Y Tanyer D. The effects of individual circadian rhythm differences on insomnia, impulsivity, and food addiction. *Eat Weight Disord.* (2019) 24:47–55. 10.1007/s40519-018-0518-x 29856005

[10] Zhang J Zhang X Zhang K Lu X Yuan G Yang H An updated of meta-analysis on the relationship between mobile phone addiction and sleep disorder. *J Affect Disord.* (2022) 305:94–101. 10.1016/j.jad.2022.02.008 35149136

[11] Huang Q Lin S Li Y Huang S Liao Z Chen X Suicidal ideation is associated with excessive smartphone use among Chinese college students. *Front Public Health.* (2021) 9:809463. 10.3389/fpubh.2021.809463 35223763 PMC8867720

[12] Park S Yang S Shin C Jang H Park S. Long-term symptoms of mobile phone use on mobile phone addiction and depression among Korean adolescents. *Int J Environ Res Public Health.* (2019) 16:3584. 10.3390/ijerph16193584 31557844 PMC6801814

[13] Cerutti R Presaghi F Spensieri V Valastro C Guidetti V. The potential impact of internet and mobile use on headache and other somatic symptoms in adolescence. A population-based cross-sectional study. *Headache.* (2016) 56:1161–70. 10.1111/head.12840 27255862

[14] Li H Zhang M Wang D Dong G Chen Z Li S Blue light from cell phones can cause chronic retinal light injury: the evidence from a clinical observational study and a SD rat model. *Biomed Res Int.* (2021) 2021:3236892. 10.1155/2021/3236892 34055970 PMC8147535

[15] Mortazavi S Mortazavi S. Women with hereditary breast cancer predispositions should avoid using their smartphones, tablets, and laptops at night. *Iran J Basic Med Sci.* (2018) 21:112–5. 10.22038/ijbms.2018.27711.6751 29456806 PMC5811748

[16] Zisapel N. Circadian rhythm sleep disorders. *CNS Drugs.* (2001) 15:311–28. 10.2165/00023210-200115040-00005 11463135

[17] Archer S Laing E Möller-Levet C van der Veen D Bucca G Lazar A Mistimed sleep disrupts circadian regulation of the human transcriptome. *Proc Natl Acad Sci U.S.A.* (2014) 111:E682–91. 10.1073/pnas.1316335111 24449876 PMC3926083

[18] Rafique N Al-Asoom L Alsunni A Saudagar F Almulhim L Alkaltham G. Effects of mobile use on subjective sleep quality. *Nat Sci Sleep.* (2020) 12:357–64. 10.2147/nss.S253375 32607035 PMC7320888

[19] Exelmans L Van den Bulck J. Bedtime mobile phone use and sleep in adults. *Soc Sci Med.* (2016) 148:93–101. 10.1016/j.socscimed.2015.11.037 26688552

[20] Touitou Y Reinberg A Touitou D. Association between light at night, melatonin secretion, sleep deprivation, and the internal clock: health impacts and mechanisms of circadian disruption. *Life Sci.* (2017) 173:94–106. 10.1016/j.lfs.2017.02.008 28214594

[21] Yin X Gou M Xu J Dong B Yin P Masquelin F Efficacy and safety of acupuncture treatment on primary insomnia: a randomized controlled trial. *Sleep Med.* (2017) 37:193–200. 10.1016/j.sleep.2017.02.012 28899535

[22] Cui C Wu L Luo F. Acupuncture for the treatment of drug addiction. *Neurochem Res.* (2008) 33:2013–22. 10.1007/s11064-008-9784-8 18618246

[23] Motlagh F Ibrahim F Rashid R Seghatoleslam T Habil H. Acupuncture therapy for drug addiction. *Chin Med.* (2016) 11:16. 10.1186/s13020-016-0088-7 27053944 PMC4822281

[24] Yang Y Li H Chen X Zhang L Huang B Zhu T. Electro-acupuncture treatment for internet addiction: evidence of normalization of impulse control disorder in adolescents. *Chin J Integr Med.* (2017) 23:837–44. 10.1007/s11655-017-2765-5 28861803

[25] Wang Y Qin Y Li H Yao D Sun B Li Z The modulation of reward and habit systems by acupuncture in adolescents with internet addiction. *Neural Plast.* (2020) 2020:7409417. 10.1155/2020/7409417 32256558 PMC7094193

[26] Kim H Cho S Park S Sohn I Jung W Moon S Can acupuncture affect the circadian rhythm of blood pressure? A randomized, double-blind, controlled trial. *J Altern Complement Med.* (2012) 18:918–23. 10.1089/acm.2011.0508 22906144 PMC3469211

[27] Wu J Chen H Chang Y Wu H Chang W Chu Y Study of autonomic nervous activity of night shift workers treated with laser acupuncture. *Photomed Laser Surg.* (2008) 27:273–9. 10.1089/pho.2007.2235 18785846

[28] Hwang D Kim H Seo J Shin I Kim D Kim Y. Sympathomodulatory effects of Saam acupuncture on heart rate variability in night-shift-working nurses. *Complement Ther Med.* (2011) 19:S33–40. 10.1016/j.ctim.2010.11.001 21195293

[29] Mullington J Abbott S Carroll J Davis C Dijk D Dinges D Developing biomarker arrays predicting sleep and circadian-coupled risks to health. *Sleep.* (2016) 39:727–36. 10.5665/sleep.5616 26951388 PMC4791606

[30] Gardner A Carpenter G So P. Salivary metabolomics: from diagnostic biomarker discovery to investigating biological function. *Metabolites.* (2020) 10:47. 10.3390/metabo10020047 PMC7073850 31991929

[31] Xiong J Zhou Z Chen W You Z Zhai Z. Development of the mobile phone addiction tendency scale for college students. *Chin Ment Health J.* (2012) 26:222–5.

[32] Driller M Suppiah H Gastin P Beaven C. Questionnaire-derived sleep habits and academic achievement in first year university students. *Clocks Sleep.* (2021) 4:1–7. 10.3390/clockssleep4010001 35076483 PMC8788481

[33] Singh S. Simple random sampling. In: Singh S editor. *Advanced Sampling Theory with Applications: How Michael ‘ Selected’ Amy Volume I.* Dordrecht: Springer (2003). p. 71–136.

[34] Hughes M Hogenesch J Kornacker K. JTK_cycle: an efficient nonparametric algorithm for detecting rhythmic components in genome-scale data sets. *J Biol Rhythms.* (2010) 25:372–80. 10.1177/0748730410379711 20876817 PMC3119870

[35] Mei S Hu Y Wu X Cao R Kong Y Zhang L Health risks of mobile phone addiction among college students in China. *Int J Ment Health Addict.* (2022). 1–16. 10.1007/s11469-021-00744-3

[36] Osadchiy V Labus J Gupta A Jacobs J Ashe-McNalley C Hsiao E Correlation of tryptophan metabolites with connectivity of extended central reward network in healthy subjects. *PLoS One.* (2018) 13:e0201772. 10.1371/journal.pone.0201772 30080865 PMC6078307

[37] Li H Xu W Jiang L Gu H Li M Zhang J Lipidomic signature of serum from the rats exposed to alcohol for one year. *Toxicol Lett.* (2018) 294:166–76. 10.1016/j.toxlet.2018.05.011 29758358

[38] Liu J Esmail F Li L Kou Z Li W Gao X Decreased frontal lobe function in people with internet addiction disorder. *Neural Regen Res.* (2013) 8:3225–32. 10.3969/j.issn.1673-5374.2013.34.006 25206643 PMC4146181

[39] Gehrman P Sengupta A Harders E Ubeydullah E Pack A Weljie A. Altered diurnal states in insomnia reflect peripheral hyperarousal and metabolic desynchrony: a preliminary study. *Sleep.* (2018) 41:zsy043. 10.1093/sleep/zsy043 29522222 PMC5946940

[40] Dallmann R Viola A Tarokh L Cajochen C Brown S. The human circadian metabolome. *Proc Natl Acad Sci U.S.A.* (2012) 109:2625–9. 10.1073/pnas.1114410109 22308371 PMC3289302

[41] Pinkofsky H Hahn A Campbell F Rueda J Daley D Douaihy A. Reduction of opioid-withdrawal symptoms with quetiapine. *J Clin Psychiatry.* (2005) 66:1285–8. 10.4088/jcp.v66n1011 16259542

[42] Fan Y Ya E Ji-Dong W Yu-Fan L Ying Z Ya-Lun S Comparison of microbial diversity and composition in jejunum and colon of the alcohol-dependent rats. *J Microbiol Biotechnol.* (2018) 28:1883–95. 10.4014/jmb.1806.06050 30270610

[43] Rehni A Singh I Singh N Bansal N Bansal S Kumar M. Pharmacological modulation of leukotriene D attenuates the development of opioid dependence in a mouse model of naloxone-induced opioid withdrawal syndrome. *Eur J Pharmacol.* (2008) 598:51–6. 10.1016/j.ejphar.2008.09.025 18840427

[44] Cajochen C Kräuchi K Wirz-Justice A. Role of melatonin in the regulation of human circadian rhythms and sleep. *J Neuroendocrinol.* (2003) 15:432–7. 12622846 10.1046/j.1365-2826.2003.00989.x

[45] Berra B Rizzo A. Melatonin: circadian rhythm regulator, chronobiotic, antioxidant and beyond. *Clin Dermatol.* (2009) 27:202–9. 19168001 10.1016/j.clindermatol.2008.04.003

[46] Jia S Guo X Chen Z Li S Liu X. The roles of the circadian hormone melatonin in drug addiction. *Pharmacol Res.* (2022) 183:106371. 10.1016/j.phrs.2022.106371 35907435

